# Curcumin Protects against Ovariectomy-Induced Bone Changes in Rat Model

**DOI:** 10.1155/2012/174916

**Published:** 2012-09-25

**Authors:** Farida Hussan, Nawwar Ghassan Ibraheem, Taty Anna Kamarudin, Ahmad Nazrun Shuid, Ima Nirwana Soelaiman, Faizah Othman

**Affiliations:** ^1^Department of Anatomy, Faculty of Medicine, Universiti Kebangsaan Malaysia, Jalan Raja Muda Abdul Aziz, 50300 Kuala Lumpur, Malaysia; ^2^Department of Pharmacology, Faculty of Medicine, Universiti Kebangsaan Malaysia, Jalan Raja Muda Abdul Aziz, 50300 Kuala Lumpur, Malaysia

## Abstract

Osteoporosis is a metabolic disease affecting both men and women especially in postmenopausal women. Curcumin possesses many medicinal properties. In this study, thirty two female Sprague-Dawley rats were used to determine the potential effect of curcumin in prevention of bone loss following ovariectomy. The animals were divided into Sham group, ovariectomised control, ovariectomised treated with curcumin 110 mg/kg and ovariectomised treated with Premarin 100 **μ**g/kg. The treatments were given via daily oral gavages for 60 days. The structural parameters such as bone volume, trabecular number, trabecular thickness and trabecular separation were found to be deteriorated in ovariectomised rats compared to Sham group. Moreover, the reduced osteoblast count, the increased osteoclast count and increased eroded surface were found in ovariectomised groups. Treatment with curcumin was able to reverse all these ovariectomy-induced deteriorations. Curcumin treatment was as effective as Premarin in most parameters except the bone volume and eroded surface, which were better than Premarin. The high dose of curcumin treatment was not only able to reduce the osteoclast number but also increase the osteoblast count. Therefore, the potential effect of curcumin can be applied as an alternative to oestrogen for prevention of postmenopausal osteoporosis.

## 1. Introduction 

Osteoporosis is a metabolic bone disorder that causes fracture in 40% of white women [1]. Bone metabolism is influenced by several factors. Bone fragility is based on the genetic and environmental factors [2]. After menopause, bone formation rate is less than bone resorption rate leading to the bone-remodelling imbalance which is associated with increased risk of fracture [3, 4]. Thus, oestrogen plays a role in bone metabolism. It controls bone resorption by reducing the osteoclast number [5]. Lack of oestrogen accelerates bone loss causing trabecular thinning and discontinuation as well as cortical thinning and porosity [2]. Estai et al. [6] found a significant reduction in trabecular number and widening of trabecular spaces in the distal portion of rats' femur 6 weeks after bilateral ovariectomy. The possible mechanism of bone loss in ovariectomised rats might be due to oxidative stress produced by the high hydrogen peroxide and lipid peroxidation levels and reduced antioxidant enzyme activities [7].

Hormone replacement therapy (HRT) reduces the fracture risk during menopause. HRT by oestrogen alone increases the risk of endometrial and breast cancer [8, 9]. The doses required for HRT in the prevention of bone loss are higher than those required in treating menopausal symptoms [3], thus the physician should outweigh the benefits and risks of HRT. Therefore, many researchers are interested in medicinal properties of natural herbs with fewer side effects.

Turmeric is a rhizome of *Curcuma longa* plant belonging to the Zingiberaceae family and Curcuma genus [10]. It is used as a spice and flavouring agent in the preparation of Asian cuisine. Curcumin (C_21_H_20_O_6_) is generally regarded as the most active constituent of polyphenolic phytochemicals, comprising 2–8% of most turmeric preparations [11]. Curcumin is a lipid-soluble active constituent which possesses varieties of potential benefits such as inhibition of lipid peroxidation in renal cell line [12], liver microsomes, erythrocyte membranes, and brain homogenates [13]. It also has anti-inflammatory [14], antimutagenic, and antihypercholesterolemia activities [15]. It has a potential protective medroxyprogesterone acetate- (MPA-) induced mammary tumours by inhibiting the expression of vascular endothelium growth factor (VEGF), *in vitro *and* in vivo* studies [16, 17]. However, it did not alter the oestrogen and progesterone receptors [16, 17]. It is able to inhibit bone resorption by stimulating the osteoclast apoptosis *in vitro* as a nuclear factor-*κ*B (NF-*κ*B) binding site competitor [18]. It also inhibited the osteoclast differentiation and function by inhibiting the signalosome-associated kinase I*κ*B in a dose-dependent response [19]. Curcumin was found to produce beneficial changes in bone turnover and bone strength [20]. The earlier study done by Anna et al. [21] found that an oral dose of 110 mg/kg body weight of curcumin protected against collagen-induced arthritis. Considering the beneficial effects of curcumin, this study was aimed to highlight the protective effect of curcumin with the dose of 110 mg/kg on bone loss due to oestrogen deficiency following ovariectomy. 

## 2. Material and Methods

### 2.1. Experimental Protocol

A total of 32 three-month-old female Sprague-Dawley rats (200–250 gm) were obtained from the Animal House after getting approval from the Animal Ethics Committee of the university. The rats were kept one rat per cage at room temperature with 12-hour light and dark cycle. The rats were allowed to access water *ad libitum* and standard rat chow (Gold Coin, Selangor, Malaysia). After one week of acclimatization, the rats were randomly divided into four groups with eight rats in each group, namely, sham-operated and given vehicle (sham), ovariectomised-control (OVXC), ovariectomised and treated with curcumin (110 mg/kg) (OVX + CL) [21], and ovariectomised and treated with Premarin (100 *μ*g/kg) (OVX + E_2_) [6]. Both curcumin and Premarin were given via oral gavage daily for 60 days. Curcumin was purchased from herbal supplier Sabinsa Company Malaysia. Palm oil without vitamin E (Merck, Germany) was used as vehicle. The 110 mg/kg dose of curcumin was freshly prepared in 1.0 mL of palm oil [21]. The treatment was started two weeks after ovariectomy. Body weights were recorded twice weekly.

### 2.2. Preparation of Oestrogen Deficient Animal Model

The rats were ovariectomised according to Estai et al. [6] under anaesthesia with intramuscular injection of combination of xylazil (0.03 mL), ketapex (0.1 mL), and zoletil 50 (0.1 mL) (Troy Laboratories, Australia). Both ovaries were removed through midline abdominal incision. Proper postoperative care was given by daily dressing with povidone iodine and treated with antibiotic enrofloxacin (Bayer, Korea) 5% intramuscularly for 7 days [6]. The same procedure was performed on the sham animals by gentle manipulation of ovaries in situ. Histology of excised tissue and marked atrophy of the uterine horns at the end of experiment confirmed the success of the surgery. 

### 2.3. Bone Histomorphometry

After 60 days of treatment, the rats were euthanized and the femora dissected out. The distal portions of the femur were kept in 10% formalin for 48 hours. The samples for structural histomorphometric analysis such as bone volume per tissue volume (BV/TV), trabecular thickness (TbTh), trabecular number (TbN), and trabecular separation (TbSp) were dehydrated and embedded in methyl methacrylate according to Difford (1974) [22]. The tissue was sectioned at 10 *μ*m with a microtome (Leica, Wetzlar, Germany) and stained with Von Kossa stain. For the static parameters such as osteoclast number (OcN), osteoblast number (ObN), eroded surface (ES), osteoid surface (OS), and osteoid volume (OV), bone samples were decalcified with EDTA for 4 weeks and embedded in paraffin wax. The samples were sectioned at 8 *μ*m with a microtome (Leica, Wetzlar, Germany) and stained with hematoxylin and eosin. The measurement for static parameters was done by using the Weibel Technique, a quantitative stereological technique for histological sections [23]. The analysis of all parameters was performed at the metaphyseal region, where the trabeculae are found abundantly [24]. True colour Windows image processing analysis system (R&M Biometrics, BQTCW98, and Version 3.50.6) interfaced with a light microscope (Olympus, Leeds Instruments, USA) was used to analyse the samples. The structural and static parameters were measured in accordance with the guidelines of the American Society of Bone Mineral Research Histomorphometry Nomenclature Committee [25]. Double-blinded assessment was done on three sections per specimen with an interval of 10 serial sections. 

### 2.4. Statistical Analysis

The results were expressed as mean values ± SD. Data analysis was performed using SPSS version 12.0. Statistical test ANOVA followed by Tukey's HSD (Honestly Significantly Different) was used for normally distributed data and Kruskal-Wallis and Mann-Whitney tests for data that was not normally distributed. The significant value was set at *P* < 0.05.

## 3. Results

### 3.1. Structural Parameters

**Figure 1:**
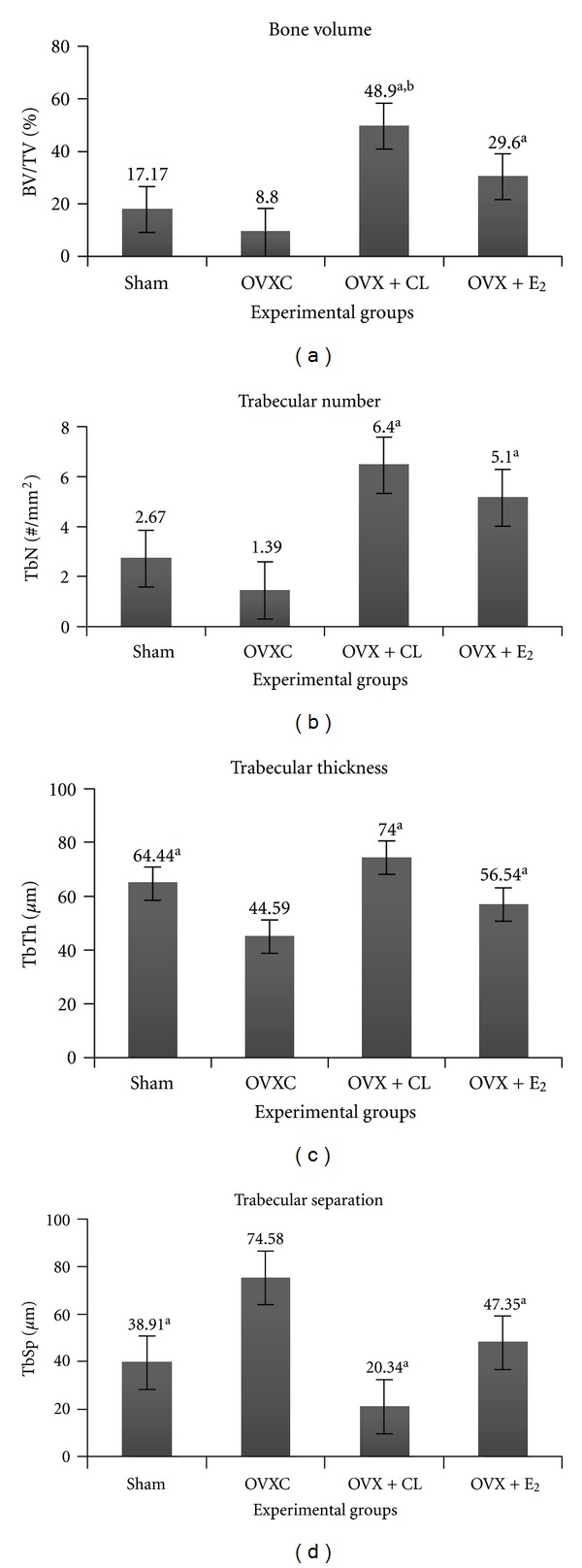
Effect of curcumin on the (a) bone volume, (b) trabecular number, (c) trabecular thickness, and (d) trabecular separation. ^a^Significant difference compared to ovariectomised (OVXC) group (*P* < 0.05). ^b^Significant difference compared to OVX + E_2_ (*P* < 0.05).

**Figure 2:**
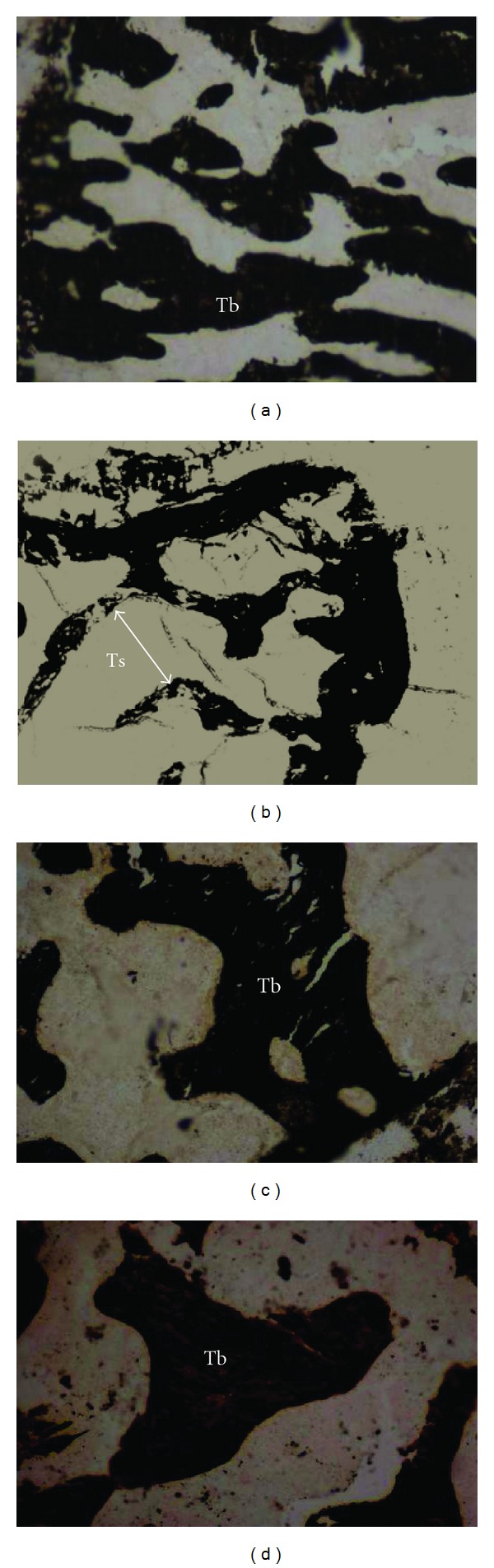
Bone trabeculae (Tb) of rat femur with Von Kossa stain 100x. (a) Sham group without treatment, (b) ovariectomised (OVX) control group—trabecular thinning and widening of trabecular space 60 days after ovariectomy, (c) OVX with curcumin—restoration of the trabecular thickness in ovariectomised rats, and (d) OVX with Premarin. Tb: trabeculae; Ts: trabecular separation.

The percentages of bone volume per tissue volume (BV/TV %) in curcumin-treated ovariectomised rats (OVX + CL) and the oestrogen replacement groups (OVX + E_2_) were significantly higher than the untreated ovariectomised groups (OVXC) (*P* < 0.05). When the two treatment groups were compared, the BV/TV of the OVX + CL group was significantly higher than that of the OVX + E_2_ (Figure 1(a)). The TbN and TbTh of OVX + CL and OVX + E_2_ groups were significantly higher than the OVXC group but were not significantly different from each other (Figures 1(b) and 1(c)). The trabecular separation (TbSp, *μ*m) was significantly higher in the OVXC group compared to the rest (Figure 1(d)). The trabeculae bony thickness of the Von Kossa stain in the OVX + CL and OVX + E_2_ (Figures 2(c) and 2(d)) were more pronounced compared to the OVXC group (Figure 2(b)). This accounted for the trabecular thinning and widening of the trabecular space in the OVXC group (Figure 2(b)) which was due to the process of osteoclastosis resulting in bone resorption. 

### 3.2. Static Parameters

**Figure 3:**
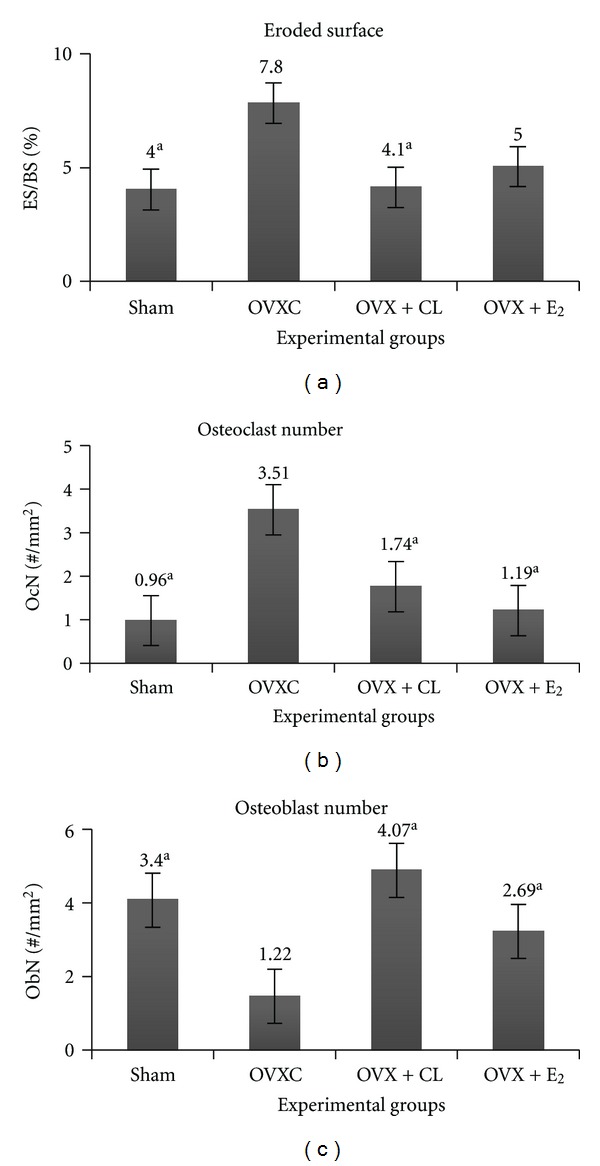
Effect of curcumin on the (a) Eroded surface, (b) osteoclast number, and (c) osteoblast number. ^a^Significant difference compared to ovariectomised (OVXC) group (*P* < 0.05).

**Figure 4:**
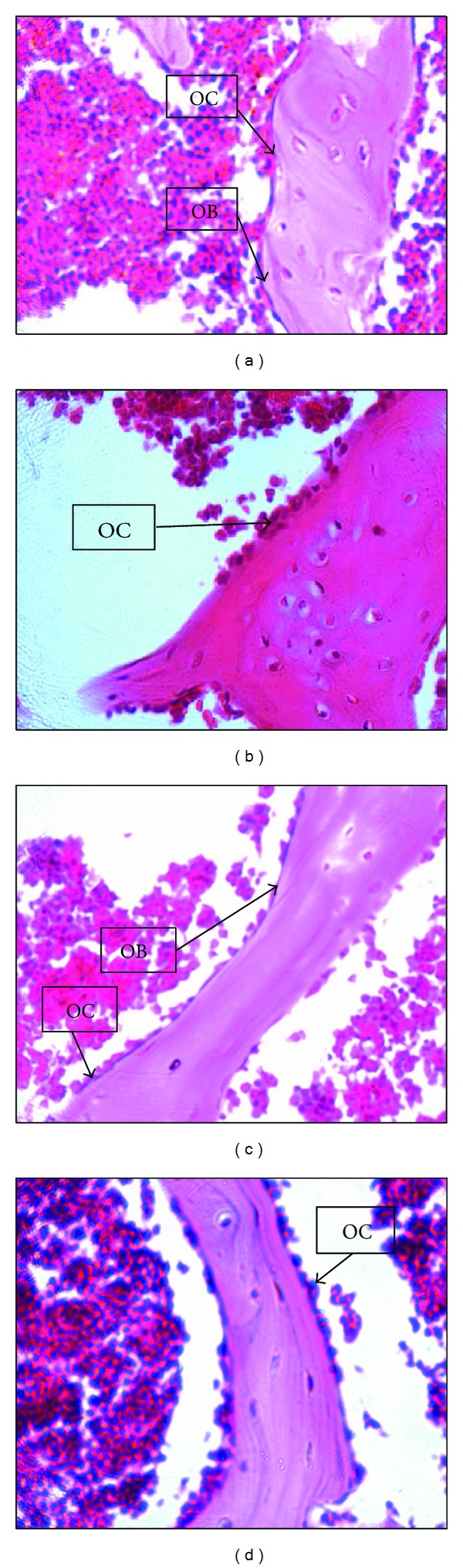
Effect of curcumin on the histological changes of bone in ovariectomised rats H&E stain 40x. (a) Sham group without treatment, (b) ovariectomised (OVX) control group, (c) OVX with curcumin, and (d) OVX with Premarin. OB: osteoblast; OC: osteoclast.

As for the static parameters of the OVXC group, the ES/BS (Figure 3(a)) and OcN (Figure 3(b)) were significantly higher while the ObN (Figure 3(c)) was significantly lower than the sham group. Both the CL and oestrogen treatments were able to significantly reduce the OcN (Figure 3(b)) and increase the ObN (Figure 3(c)) compared to sham group. Only treatment with CL was able to reduce the ES/BS (Figure 3(a)) until it was significantly lower than the OVXC group. The OS and OV of all the groups were not significantly different from one another (data not shown). In the H&E stain, indentations of the Howship's lacunae with numerous multinucleated osteoclasts were observed in the OVXC and OVX + E_2_ group (Figures 4(b) and 4(d)). These were less obvious in the sham and OVX + CL groups (Figures 4(a) and 4(c)). Flattened-looking osteoblasts were observed laid on the bony surfaces of the sham and the OVX + CL groups indicating some form of ossification (Figures 4(a) and 4(c)). 

## 4. Discussion

World Health Organisation (WHO) study group [3] suggested that the definition of osteoporosis should be retained as “A disease characterised by low bone mass and microarchitectural deterioration of bone tissue, leading to enhanced bone fragility and a consequent increase in fracture risk.” This definition was developed during the Consensus Development Conference in 1991 [26]. Skeletal fragility and fracture risk are associated with low bone mineral density (BMD) [27]. Therefore, BMD is used as an indicator to measure bone mass and serves as a diagnostic parameter for osteoporosis [28]. A study group of WHO [3] reported that the increased fracture risk is associated with BMD as low as the *T*-score of ≤2.5 SD. 

In normal individuals, bone mass increases during skeletal growth and reaches the peak bone mass between the ages of 20 to 40 years. Postmenopausal osteoporosis occurs due to low peak bone mass or accelerated bone loss due to hormone deficiency or aging or both factors. However, the mineral content of the remaining bone could be normal and thus there is no shift in the ratio of minerals to protein matrix [29]. The most important microscopic features of bone loss include widening of Haversian canals and thinning of the trabeculae that could be due to increased bone resorption and perforation of trabecular plates [30].

In oestrogen deficiency state, there were reductions in trabecular number (TbN) and bone volume (BV/TV) and increased trabecular separation (TbSp) which are attributed by the higher bone resorption than bone formation [31]. Estai et al. [6] also mentioned that the trabecular numbers were reduced as early as the 6th week after ovariectomy in young female Sprague Dawley rats. The present study revealed similar findings in ovariectomised control rats by the 10th week after ovariectomy. This was in accordance with the observation by Baldock et al. [24] which found that the trabecular bone loss in ovariectomised rat was mainly due to the decrease in trabecular number. 

Oestrogen deficiency induces bone loss causing trabecular thinning [2]. This is explained by the fact that lack of oestrogen stimulates the differentiation and the proliferation of osteoclasts [32]. However, the previous study [6] showed no significant change in trabecular thickness till the 6th week following ovariectomy. In the present study, the trabecular thickness was significantly reduced by the 10th week after ovariectomy. The present finding supported the fact that oestrogen deficiency accelerates bone resorption [5]. 

However, these ovariectomy-induced bone changes were reversed with curcumin treatment. The present study found that the bone structural changes were significantly reversed in curcumin-treated ovariectomised group compared to ovariectomised-control group. Our results showed that curcumin extract was able to protect the trabecular bone volume against the effect of ovariectomy. These were in agreement with Hie et al. [33], who found that curcumin reduced diabetes-associated bone resorption. Tuba and Gülçin [34] reported that curcumin possesses free radical scavenging activity. This beneficial property may apply to its protective effect against bone loss due to oestrogen deficiency that induces oxidative stress which stimulates the differentiation and proliferation of osteoclasts via cytokine release [32]. 

The increased osteoclast count in ovariectomised group of the present study supported the findings of Parhami [32]. Ozaki et al. [18] had examined the action of curcumin on rabbit osteoclast apoptosis and suggested that curcumin may be useful in the treatment of osteoporosis as it drastically inhibited bone resorption in parallel with stimulation of apoptosis of the cells. At the molecular level, macrophage colony-stimulating factor (M-CSF) and RANKL, a receptor activator of NF-*κ*B ligand, are essential for differentiation of osteoclasts, and responsible for bone resorption [35]. Kim et al. [36] recently reported that curcumin inhibits osteoclastogenesis by impairing the signalling of RANKL. Therefore, curcumin may have affected the activity and number of osteoclasts rather than osteoblasts in the bone of diabetic rats [33]. This was in agreement with the present study which found reduction in osteoclast number of ovariectomised rats with curcumin treatment. 

Another important consideration for curcumin treatment is the dosage, duration, and mode of administration. Curcumin was found to induce bone changes after ovariectomy in a dose-dependent manner [20]. The dose of 110 mg/kg body weight used in the present study which was administered by oral gavage is considered high compared to previous studies [20, 33]. However, the dose used was claimed to be safe as there have been no reports of significant adverse effects with the consumption of 500 to 8,000 mg turmeric powder per day in human [37, 38]. Our findings suggested that higher dose of curcumin not only inhibited the osteoclast activity but was also able to stimulate the activity of osteoblast, the cells responsible for osteoid synthesis and mineralisation of matrix [39]. 

Oestrogen is important in maintaining bone metabolism by inhibiting the osteoclast activity. Premarin, a conjugated oestrogen, is used for the prevention of postmenopausal osteoporosis. Although it is effective in alleviating the postmenopausal syndrome, there are higher risks of endometrial and breast cancers to women with strong family histories. The Woman Health Initiative Study Group [40] reported that it may also increase the risk of stroke and deep vein thrombosis following long-term use because oestrogen induces clotting factors release from the liver. However, curcumin delays breast cancer development related with combined hormonal therapy [17]. It was reported to inhibit the tissue factor activity implicated in thrombotic disorders [41] and decreased platelet adhesion and activation [42]. These actions were achieved through its anti-inflammatory, anticarcinogenic, and antiproliferative effects [41]. Therefore, curcumin reduced the risk of adverse effects of oestrogen and, at the same time, revealed the inhibitory effect on the bone changes following ovariectomy. Its protection on bone changes was comparable to the oestrogen as proven in the present study. 

## 5. Conclusion

Curcumin treatment reversed the bone changes following ovariectomy and it was as effective as oestrogen therapy. Further studies are warranted to explore the potential of curcumin as an alternative agent for oestrogen in postmenopausal osteoporosis.
